# COVID‐19 and Cognitive Decline: AI‐Driven Insights into Risk Detection Among Older Adults using All of Us Dataset

**DOI:** 10.1002/alz70861_108255

**Published:** 2025-12-23

**Authors:** Zahra Rahemi, Mohadeseh Hashemi, Meisam Omidi

**Affiliations:** ^1^ Clemson University, Greenville, SC USA; ^2^ Axle Informatics, La Marque, TX USA; ^3^ Marquette University, Milwaukee, WI USA

## Abstract

**Background:**

COVID‐19 has introduced new challenges in dementia research, with growing evidence suggesting a potential role in accelerating cognitive decline. However, it remains unclear whether COVID‐19 acts as an independent risk factor or amplifies existing vulnerabilities. This study applies AI‐enhanced analysis to investigate post‐COVID cognitive decline and its associated health patterns. These findings contribute to the development of predictive models that integrate infection history and clinical risk profiles to support early detection and risk stratification.

**Methods:**

Using retrospective data from 26,952 individuals aged 50 and older with confirmed COVID‐19, we defined a study group (*n* =1,721) with new cognitive impairment diagnoses within 30 days post‐infection and a control group (*n* =25,231) without such diagnoses. In Phase 1, we employed unsupervised learning techniques, including clustering and association rule mining, to identify comorbidity patterns within the study group. Phase 2 applied logistic regression and supervised learning models (Random Forest, XGBoost), incorporating SHAP values for model interpretability. All analyses were adjusted for age, sex, comorbidities, education, and selected social and behavioral factors available in the dataset.

**Results:**

COVID‐19 severity was independently associated with an increased risk of post‐infection cognitive impairment after adjusting for age, sex, comorbidities, and socioeconomic factors (adjusted OR > 2.0, *p* < 0.001). Clustering analysis identified high‐risk subgroups defined by co‐occurring cardiometabolic, neuropsychiatric, and inflammatory conditions, which contributed to higher risks of cognitive impairment. Supervised machine learning models achieved strong predictive performance (AUC > 0.85), with key predictors including age, depression, and education level. Differences in risk patterns across the study and control subgroups suggest the utility of adaptable modeling strategies to support individualized risk estimation.

**Conclusion:**

COVID‐19 appears to be a significant driver of cognitive impairment in older adults, particularly among individuals with specific comorbid profiles. Our findings reinforce the importance of integrating infection history, comorbidities, and individual background factors into models of cognitive decline risk. This study supports the development of explainable AI tools that model varied cognitive trajectories and inform individualized intervention planning across a range of patient profiles. Future work will focus on refining these models for clinical integration and validating their utility in prospective real‐world settings.

